# Associations Between Resilience, Psychological Well-Being, Work-Related Stress and Covid-19 Fear in Forensic Healthcare Workers Using a Network Analysis

**DOI:** 10.3389/fpsyt.2021.678895

**Published:** 2021-06-11

**Authors:** Stefan Bogaerts, Marianne van Woerkom, Yasemin Erbaş, Elien De Caluwé, Carlo Garofalo, Iris Frowijn, Ingeborg Jeandarme, Erik Masthoff, Marija Janković

**Affiliations:** ^1^Department of Developmental Psychology, Tilburg University, Tilburg, Netherlands; ^2^Fivoor Academy of Research, Innovation and Development (FARID), Rotterdam, Netherlands; ^3^Department of Human Resource Studies, Tilburg University, Tilburg, Netherlands; ^4^Department of Psychology, Education and Child Studies, Erasmus University Rotterdam, Rotterdam, Netherlands; ^5^Department of Criminal Law and Criminology, Katholieke Universiteit Leuven, Leuven, Belgium; ^6^Knowledge Center Forensic Psychiatric Care, Openbaar Psychiatrisch Zorgcentrum Rekem, Rekem, Belgium

**Keywords:** forensic healthcare workers, psychological well-being, work-related stress, resilience, COVID-19 pandemic

## Abstract

Forensic healthcare workers deal with patients with severe psychiatric and behavioral problems that put them at an increased risk of developing work-related stress and burnout. Working with this target group of patients during the Coronavirus disease 2019 (Covid-19) pandemic with far-reaching restrictive measures can negatively affect the psychological well-being of forensic workers. Research suggests that resilience can buffer workplace stress and contribute positively to psychological well-being. However, research on resilience, psychological well-being and work-related stress among forensic healthcare workers is still lacking. Therefore, in this study, we investigated the interrelations between psychological well-being and resilience on the one hand and work-related stress and Covid-19 fear-related symptoms on the other hand. Self-report data were obtained from 318 healthcare workers (73.9% women) working in three Forensic Psychiatric Centers (*M age* = 44.20, *SD* = 14.31) and are in direct contact with forensic patients. The data were analyzed using network analysis. Consistent with previous research, the results showed that workplace stress and fear associated with the Covid-19 pandemic can be detrimental to workers' psychological well-being, while resilience can serve as a protective factor against being personally attacked or threatened by patients at the workplace. Last but not least, we identified highly central symptoms, namely *tremors due to the fear of the coronavirus* and *anxiety when other people coughing*, which would be the best candidates for future treatment targets. This knowledge can help clinicians optimize interventions to reduce workplace stress and fear due to the pandemic. Future studies should aim to replicate our findings in a larger and more representative sample of forensic healthcare workers.

## Introduction

Working in healthcare settings is an emotionally demanding job that can negatively affect well-being. Notably, research on well-being of healthcare workers shows that psychological well-being and resilience can counter the adverse effects of stressful work-related life events (1). So far, this research has not been conducted in a forensic context. Yet, working with forensic patients is highly demanding due to the nature of the population and their specific behavioral characteristics. Indeed, research has shown that workplace aggression is significantly more common in forensic psychiatry compared to general psychiatry (2). Therefore, forensic healthcare workers may be particularly at risk for lower psychological well-being (3), hence being more vulnerable to the effects of stressful work-related life events. Otherwise, psychological well-being and resilience can counter job-related stress. The present study was conducted during the Covid-19 crisis, which is an additional stress factor for forensic healthcare workers. In this research, we focus on work-related stress, psychological well-being, resilience, and psychosomatic and social factors related to the fear of Covid-19 in a group of forensic mental healthcare workers.

Forensic healthcare workers can experience emotional burden because forensic patients often have committed criminal offenses that are violent in nature, and exhibit behavioral problems within the forensic setting (4, 5). The risk of derailment that can lead to verbal and physical inpatient violence is always present. In addition, most aggressive outbreaks are unpredictable and can be triggered by personal factors (e.g., psychopathology), environmental factors (e.g., tensions in the living group), and the interaction between personal and environmental factors (6). Hence, the forensic healthcare worker is constantly in a state of alertness (2). Besides, forensic patients are often not intrinsically motivated to change deviant behavior, thereby hindering their susceptibility to forensic treatment and potentially increasing the frustration of staff (7). These aspects may cause conflicts between patients and healthcare staff, which sometimes lead to restrictive measures, such as temporary removal from the living group or the suspension of leave modalities (8). Taken together, the specific circumstances associated with working as a healthcare professional in forensic care settings can cause work pressure and stress (9–11), thereby reducing productivity and negatively affecting psychological well-being.

The Jobs Demands-Resources Model underlines the importance of a balance between high job demands and available job or personal resources (12). When job demands are high, a lack of resources can lead to stress response and health problems (exhaustion process), while sufficient resources can lead to high motivation and productivity (motivational process). Because working with forensic patients is highly demanding, it requires personal resources to perform the job optimally (12).

Psychological well-being of healthcare workers refers to the positive emotional and psychological functioning of individuals and is an important aspect of mental health. Research has shown that occupational stress can have a large impact on psychological well-being (13).

An important factor that can protect individuals' well-being from the negative effects of work-related stress on a personal and organizational level is resilience [e.g., (14, 15)]. Resilience can be conceptualized as personality and cognitive traits of self-confidence, resourcefulness, curiosity, self-discipline, sobriety, and flexibility along with problem-solving skills and emotional stamina (16, 17). It can also be defined as the ability to bounce back and recover from a bad day or setback (18–22). A systematic review shows that resilience can buffer against emotional exhaustion and burnout among nurses working in healthcare settings (23).

A consistent finding in many studies is that resilience is positively associated with general well-being and negatively associated with work-related stress, anxiety and post-traumatic stress. Resilience has been found to play a mediating role in the relationship between work-related stress and general well-being (23). Individual differences in burnout are related to individual differences in resilience and the development of burnout symptoms can be mitigated and reversed when resilience is strong (24). Resilience can also be trained through short, targeted interventions that can support healthcare workers in learning to deal with stressful work-related factors. Given the high job demands of forensic healthcare workers and the importance of understanding the resilience of forensic workers, it is necessary to investigate whether resilience can protect against work-related stress and Covid-19 fear-related factors, and contribute to greater psychological well-being. However, despite more extensive research on nursing staff in general healthcare settings, research on resilience, psychological well-being and work-related stress among forensic healthcare workers is still lacking.

The current study took place during the Covid-19 pandemic, in the period between June and July 2020. This means that on top of working with a difficult patient group, forensic healthcare workers also had to deal with the Covid-19 pandemic and the measures that were taken. Several measures were implemented that have altered working environments, such as mandatory lockdowns, social distancing measures, wearing masks, learning new techniques and skills related to hygiene, disinfection, telemedicine, quarantining of admission, and fewer therapeutic sessions with patients (25). At the time of the survey, there was a relaxation of the restrictive Covid-19 measures in both the Netherlands and Belgium. Forensic patients were allowed to see external visitors again and leave provisions were restarted. However, research shows that the Covid-19 pandemic can disrupt people's routines and can provoke fear and phobic responses (26). Corona fear can be described as a persistent and excessive fear of the coronavirus, which can be classified as a particular type of the *DSM-5* specific phobia (27). The main characteristic of specific phobias is fear or anxiety related to the source of the phobia. Serious negative psychosomatic (e.g., physical complaints) and social effects (e.g., fear of coughing people) of the Covid-19 pandemic, as have been observed in many countries, can have a negative impact on psychological well-being [e.g., (26, 28)]. The impact of Covid-19 and the national lockdowns, on top of the stress-evoking factors associated with working in a forensic environment can make forensic healthcare workers extra vulnerable for developing mental health problems. Therefore, when examining links between work-related stress, psychological well-being, and resilience in this specific historical phase, it is imperative to take into account the specific impact of Covid-19 and related fears.

Because of the assumption that work-related stress, resilience, psychological well-being, as well as Covid-19 psychosomatic and social variables might be mutually interrelated in a complex way, it is important to obtain insight into their reciprocal associations. Specifically, as we previously discussed, symptoms related to the fear of Covid-19 can exacerbate work-related stress symptoms, which in turn may adversely affect workers' well-being. Resilience may serve as a protective factor against the negative impact of these symptoms on well-being. Hence, to study these associations comprehensively, we will adopt a network approach because it provides insight to understand item-level relationships to improve psychological science and clinical practice. This approach has been already applied in forensic psychiatry (29) as well as in other areas, such as psychopathology (30), and personality research (31). Network analysis can help us to visualize and quantify complex associations between different symptoms/constructs and mental health outcomes. In psychological networks, nodes correspond to variables, while edges represent statistical relationships [e.g., (32)]. In addition, a network approach allows us to identify the most central construct (or node) in a network of associations, that is, the construct that has the strongest connection with the other constructs in the network. Arguably, according to the network approach, by addressing the most central node, it is likely that other nodes (or symptoms) will be impacted in the desired direction (e.g., symptom reduction, increased well-being). To our knowledge, there are no studies investigating associations between work-related stress, resilience, psychological well-being and Covid-19 fear in forensic healthcare workers using network analysis, although this approach may be invaluable to provide support to staff members by identifying those aspects that have the most critical impact.

In this study, we investigated work-related stress, psychological well-being, resilience and Covid-19 fear in forensic healthcare workers using a network analysis. The aim of this study was to obtain insights into the item-level associations between work-related stress and Covid-19 psychosomatic and social fear items, and the scale-level variables of psychological well-being and resilience. We expected that the items representing work-related stress and the Covid-19 psychosomatic and social fear variables on the one hand, and psychological well-being and resilience on the other hand would be strongly interrelated (26). More specifically, we expected psychological well-being and resilience to be negatively associated with the work-related stress items as well as the Covid-19 psychosomatic and social items (15). We also expected strong positive associations between psychological well-being and resilience (23). Finally, we expected that the Covid-19 fear-related symptoms would be the most central in the network compared to the work-related stress symptoms, resilience, and psychological well-being. In other words, fear associated with Covid-19 may increase stress at the workplace, which can further negatively affect well-being, while resilience may change the adverse impact of work-related stress symptoms on well-being.

## Materials and Methods

### Participants

The sample consisted of 318 healthcare workers from three Forensic Psychiatric Clinics (FPCs) of which 126 (39.6%) worked in two Belgian institutions and 192 (60.4%) in a Dutch institution. This equates to a response rate of 21.9% in total. The group of women was over-represented (*n* = 235, 73.9%) due to the fact that more women than men are working in forensic psychiatry. The average age of the healthcare workers was 44.20 years (*SD* = 14.31, range = 21–76). The largest group was married (*n* = 97, 30.5%), followed by living alone (*n* = 116, 36.5%), cohabiting (*n* = 79, 24.8%), divorced (*n* = 9, 2.8%) and widowed state (*n* = 2, 0.6%). Information was missing from one respondent. The educational background varied from high school (*n* = 28, 8.8%), intermediary or higher vocational education (*n* = 167, 52.5%) to a university degree (*n* = 119, 37.5%). Information was missing from six respondents (1.3%). For further information about the descriptive statistics (means, standard deviations), we refer to Table 1.

**Table 1:** Means and standard deviations for all variables (*n* = 318).

**Demographic characteristics**	***M* (*SD*)/*N* (%)**
Age	44.2 (14.31)
Females	235 (73.9 %)
Years in the organization	5.77 (6.64)
Working hours per week	33.96 (6.97)
**Questionnaire characteristics**	***M*** **(*****SD*****)**
Work-related stress symptoms (range 0–3)	
Is your job emotionally demanding?	1.61 (0.66)
Are you confronted in your work with things that affect you personally?	1.33 (0.60)
Do you feel personally attacked or threatened in your work?	0.80 (0.61)
Do you have contact with difficult clients or patients in your work?	2.09 (0.73)
Does your job require persuasion?	1.69 (0.75)
Does your work put you in harrowing situations?	1.51 (0.62)
Total score	10.50 (3.06)
Resilience (range 1–4)	
I have confidence in myself	3.03 (0.66)
I can easily adjust in a difficult situation	2.99 (0.69)
I am able to persevere	3.43 (0.56)
After setbacks, I can easily pick up where I left off	3.03 (0.67)
I am resilient	3.15 (0.60)
I can cope well-with unexpected problems	2.97 (0.64)
I appreciate myself	2.89 (0.73)
I can handle a lot at the same time	2.74 (0.81)
I believe in myself	3.00 (0.71)
Total score	27.24 (4.05)
Psychological well-being (range 0–5)	
I have felt cheerful in good spirits	2.86 (1.13)
I have felt calm and relaxed	2.89 (1.16)
I have felt active and vigorous	3.14 (1.18)
I woke up feeling fresh and rested	2.77 (1.28)
My daily life has been filled with things that interest me	3.01 (1.24)
Well-being index	58.77 (18.81)
Covid-19 fear-related psychosomatic symptoms (range 1–5)	
I experience severe stomachaches out of the fear of coronavirus	1.33 (0.64)
I experience serious chest pain out of the fear of coronavirus	1.25 (0.57)
I experience tremors due to the fear of coronavirus	1.21 (0.49)
I experience sleep problems because out of the fear of coronavirus	1.52 (0.87)
Coronavirus makes me so tense that I find myself unable to do the things I previously had no problem doing	1.45 (0.79)
Total score	6.78 (2.76)
Covid-19 fear-related social symptoms (range 1–5)	
After the coronavirus pandemic, I feel extremely anxious when I see people coughing	1.71 (0.83)
After the coronavirus pandemic, I actively avoid people I see sneezing	2.27 (1.17)
Following the coronavirus pandemic, I have noticed that I spend extensive periods of time cleaning my hands	2.53 (1.30)
The fear of coming down with coronavirus seriously impedes my social relationships	2.06 (1.52)
I am unable to curb my anxiety of catching coronavirus from others	1.52 (0.85)
Total score	10.06 (3.98)

### Procedure

This study is part of an ongoing project entitled “Working in Corona Times,” which investigates psychological factors affecting the mental health of forensic healthcare workers during the Covid-19 outbreak. The current study only refers to the first wave of data collection that was conducted in the period between June and July 2020. The only inclusion criterion was that participants had a treatment relationship (e.g., nurses, therapeutic assistants, psychologists, psychiatrists, music therapists, and psychomotor therapists) with forensic patients and sufficient knowledge of the Dutch language to be able to understand the questionnaires. In Belgium, questionnaires were distributed to approximately 250 forensic workers and, in the Netherlands, to ~1,200 forensic workers. This study was conducted using online questionnaires. All employees of the three institutions were informed 2 weeks in advance about the survey by the Human Resources and communication departments in collaboration with the directors and researchers. To complete the digital questionnaires, participants had to give informed consent and were informed that they could stop participating in the study at any time without giving a reason. A total of 418 employees completed the questionnaires (28.83%) of which 261 (21.75%) worked in the Dutch FPC and 157 (62.8%) worked in two Belgian FPCs. Of these 418 employees, 318 employees worked as healthcare workers with direct patient contact. One hundred employees worked in the support services (e.g., kitchen, secretariats, and support services) and were excluded because this study investigates respondents with direct patient contact (see Supplementary Figure 1). Participants spent ~20 to 30 min completing the questionnaires. The study was approved by the boards of directors of the institutions and by the Scientific Research Committee of Fivoor.

### Measures

#### Work-Related Stress

A seven-item scale that is part of the Questionnaire on the Experience and Evaluation of Work (33) was used to measure work-related stress in the past 3 months. This specific scale investigates the emotional burden of the work. Two example items are: “Do you have contact with difficult clients or patients in your work? and “Do you feel personally attacked or threatened in your work?” Respondents reported their experience of specified emotional work-related burden on a four-point scale ranging from 0 (never) to 3 (always). In our study, Cronbach's alpha was α = 0.79, indicating good internal consistency of the items in the scale.

#### Resilience

The Resilience Evaluation Scale (34) is a scale that consists of nine items with three items reflecting self-confidence (e.g., “I have confidence in myself”) and six items loading on self-efficacy (e.g., “I can easily adjust in a difficult situation”). The nine items must be answered on a five-point Likert scale ranging from “Strongly disagree” (0 points) to “Totally agree” (4 points), resulting in an average score between 0 and 4. The higher the average score, the more resilient the participant is (34). In previous research, the RES showed good convergent validity and internal consistency, and is measurement invariant across Dutch- and English-speaking groups (34, 35). In the current study, the internal consistency of the RES scale was very good with Cronbach's alpha being α = 0.84.

#### Psychological Well-Being

The World Health Organization-5 Well-Being Index (36) was used to measure psychological well-being. This five-item scale includes items such as “I have felt cheerful and in good spirits” and “My daily life has been filled with things that interest me.” Participants rated answers on a six-point Likert scale ranging from 0 (not present) to 5 (constantly present). A review study validated the methodological aspects of this questionnaire and found satisfactory validity as a screening tool in relation to an outcome measure of depression in most adapted language versions (37). Specifically, a Dutch research team validated the scale and found satisfactory psychometric properties with a Cronbach's α of.82 (38). In this study, Cronbach's alpha was α = 0.85, indicating a very good internal consistency of the WHO-5 scale.

#### Covid-19 Fear

We included two sub-dimensions (10 items) of the Covid-19 fear scale (C19P-S; 26), namely psychosomatic (five items) and social (five items) phobia. We did not include the economic and psychological dimensions as they were unrelated to the study purposes. All responses were scored on a five-point Likert scale ranging from 1 (strongly disagree) to 5 (strongly agree). An example item of the psychosomatic scale is “I experience severe stomachaches out of the fear of coronavirus”; an example item of the social fear scale is “The fear of coming down with coronavirus seriously impedes my social relationships.” In the validation study, the Cronbach's alpha of the Turkish version of C19P-S was α = 0.92 for the 20-item scale. Sub-dimension scores were obtained by the sum of the points of the answers given to the items belonging to that sub-dimension. In this study, we found a Cronbach's alpha of α = 0.86 for both scales. Both scales showed a very good internal consistency.

### Statistical Analyses

First, SPSS version 26 was used to calculate the skewness and kurtosis of the items and to determine if the distribution of the items was symmetrical and not too peaked (39). For skewness and kurtosis, stomach ache, tremors and chest pain did not meet the assumptions of normal distribution and therefore the randomization technique bootstrapping was used (40). Second, all items of the scale work-related stress and Covid-19 fear (psychosomatic and social fear) and the outcome variables psychological well-being and resilience (scale-level) were entered and analyzed in a network model. Because the number of nodes and edges in a network model must be limited to maintain sufficient power, we did not opt for a completely “scale-free” network, but included psychological well-being and resilience as composite scores (41). In total, we included two variables at scale level (psychological well-being and resilience) and 17 observed variables at item level. For an overview of the variables and abbreviations used in the network model, see Supplementary Table 1. The network structure was estimated with the Gaussian Graphical Model (31) using the R-package qgraph (42). The GGM is a network analysis technique for continuous or ordinal normally distributed data, in which nodes represent variables and the edges represent partial correlations between the items (31). More specifically, an edge represents an interaction between two variables after conditioning on all other variables in the network. We employed the Extended Bayesian Information Criterion (EBIC) model selection (43) to test for false positive edges, which resulted in a network with as few edges as possible. Generally, green edges indicate a positive association between the variables, red edges represent a negative association between the variables, and the thicker the edges, the stronger the associations (44). When analyzing network models, three indices of centrality can be distinguished, namely degree centrality, closeness centrality and betweenness centrality (45). Degree centrality refers to the sum of direct edges a node has with other nodes. The more direct edges a node has to other nodes, the more important the node is in a graph (46). Closeness centrality of a node is a measure of centrality in a network that is calculated as the normalized average of all of its geodesic distances (the shortest path between two points on a curved surface). Closeness centrality calculates the shortest paths between all nodes and assigns to each node a score based on its sum of the shortest paths (47). A node has high closeness centrality if this node can reach other nodes quickly (48). Betweenness centrality shows which nodes are bridges between nodes in a network. It is calculated by identifying all the shortest edges between all node pairs and then counting how many times each node falls on one of these paths. High betweenness nodes can have a significant influence within a network because of their control over information passed between other nodes (45). The stability of centrality indices was based on *subsetting* (49). The procedure reveals if the order of centrality measures remains the same after re-estimating the network with fewer cases. To quantify the stability of centrality indices further, the correlation stability coefficient (CS-coefficient) was calculated. It represents the maximum number of cases that can be dropped from the data while retaining a 95% probability that a correlation between estimated centrality indices and the original centrality coefficients is 0.7 or higher. We will interpret only the stable centrality measures, that is, the centrality measures with a CS-coefficient ≤ 0.25 (49). Lastly, the bootstrapped difference test was used to test if the edge weights or centrality indices differ significantly from one another using 1,000 bootstrap samples and α = 0.05. Only the edge-weights/centrality measures that were significantly higher than most other edges/centrality measures in the network were interpreted.

## Results

### Network Structure

**Figure 1:**
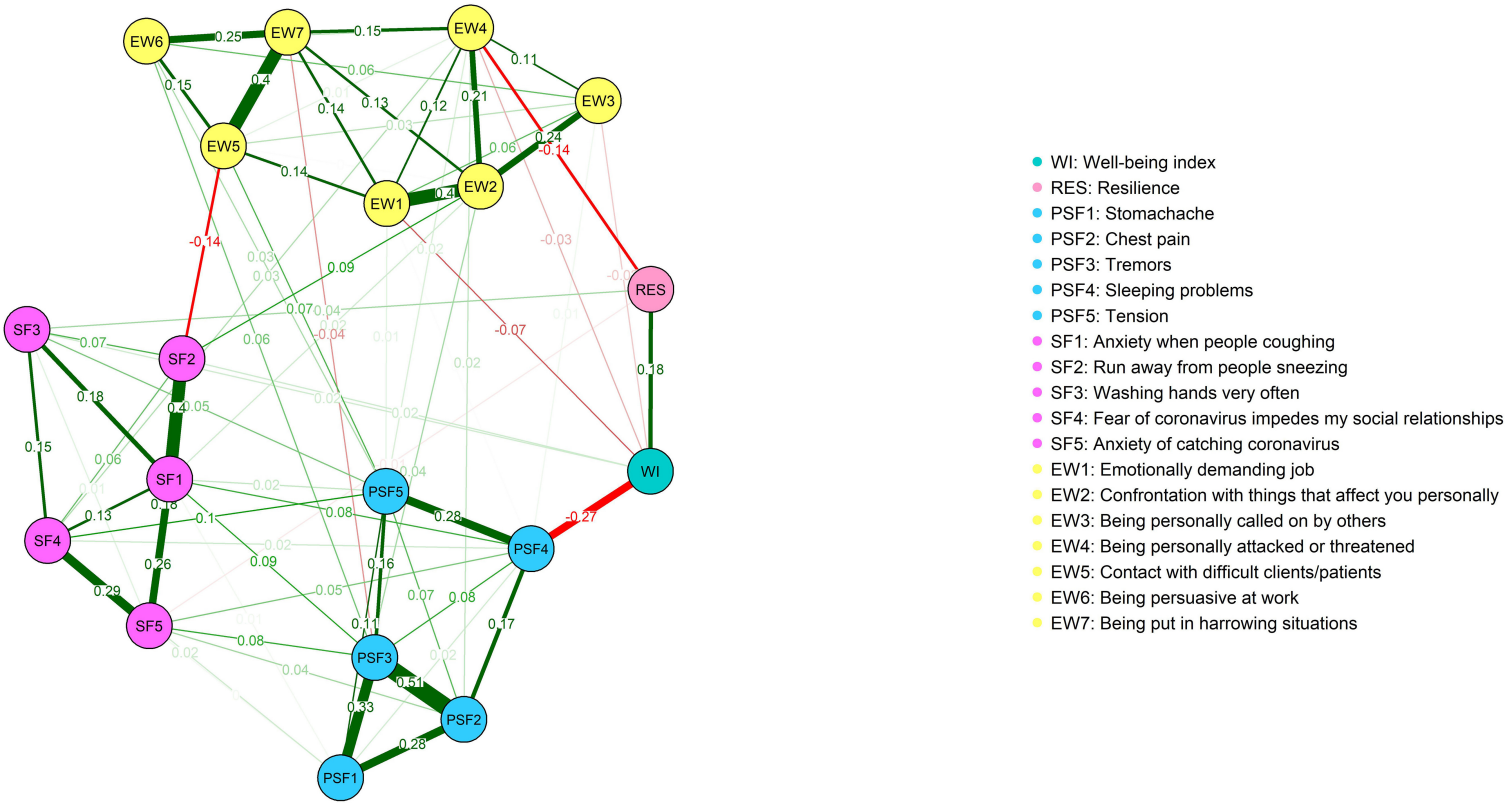
Network structure of Covid 19 fear symptoms, work-related stress, resilience, and psychological well-being.

The estimated network of individual corona-related psychosomatic and social fear symptoms, work-related stress symptoms, and resilience and psychological well-being scale scores are shown in Figure 1. Approximately 43% of all edges were non-zero edges (74/171) and 57% were zero edges (sparsity). Overall, the associations within clusters were strong and positive, meaning that the items belonging to the clusters work-related stress and covid-19 were strongly associated with each other, while those among clusters were weaker and both positive and negative.

#### Interrelations Between Resilience, Psychological Well-Being, Work-Related Stress and Covid-19 Fear

Given the associations among clusters (see Figure 1), the psychological well-being index (WI) was strongly and negatively associated with sleeping problems (PSF4), moderately and positively associated with resilience (RES), and weakly and negatively associated with emotionally demanding job (EW1). There were also two weak and negative edges between running away from sneezing people (SF2), and contact with difficult clients/patients (EW5) and between being personally attacked or threatened (EW4), and resilience (RES). These results were supported by our network parameter accuracy analysis (i.e., the bootstrapped difference test; see Supplementary Figure 2).

#### Connections Within Covid-19 Fear and Work-Related Stress Symptoms

As Figure 1 shows, the strongest edge among psychosomatic Covid-19 fear-related symptoms was between chest pain (PSF2) and tremors (PSF3). Tremors (PSF3), in turn, were strongly associated with stomachache (PSF1), while stomachache (PSF1) was moderately associated with chest pain (PSF2). It should be noted that these edges, i.e., stomachache (PSF1), chest pain (PSF2) and tremors (PSF3) formed a triad. Lastly, there was also a moderate association between sleeping problems (PSF4) and tension (PSF5). Regarding social corona fear symptoms, the strongest edge was between anxiety when people coughing (SF1) and run away from people sneezing (SF2). People's fear of coughing (SF1) was also moderately associated with anxiety of catching the coronavirus (SF5), and somewhat less strongly associated with washing hands very frequently (SF3) and fear of the coronavirus hindering social relationships (SF4), respectively (for the bootstrapped difference test, see Supplementary Figure 2).

Considering work-related stress symptoms (see Figure 1), the strongest edges were between emotionally demanding job (EW1) and confrontation with things that affect you personally (EW2), and between contact with difficult clients/patients (EW5), and getting in harrowing situations (EW7). The confrontation with matters that affect you personally (EW2) was also moderately associated with being personally called on by others (EW3) and being personally attacked or threatened (EW4), respectively. Finally, there was also a weak edge between contact with difficult clients/patients (EW5) and persuasiveness at work (EW6) (for the bootstrapped difference test, see Supplementary Figure 2).

### Centrality Indices

**Figure 2:**
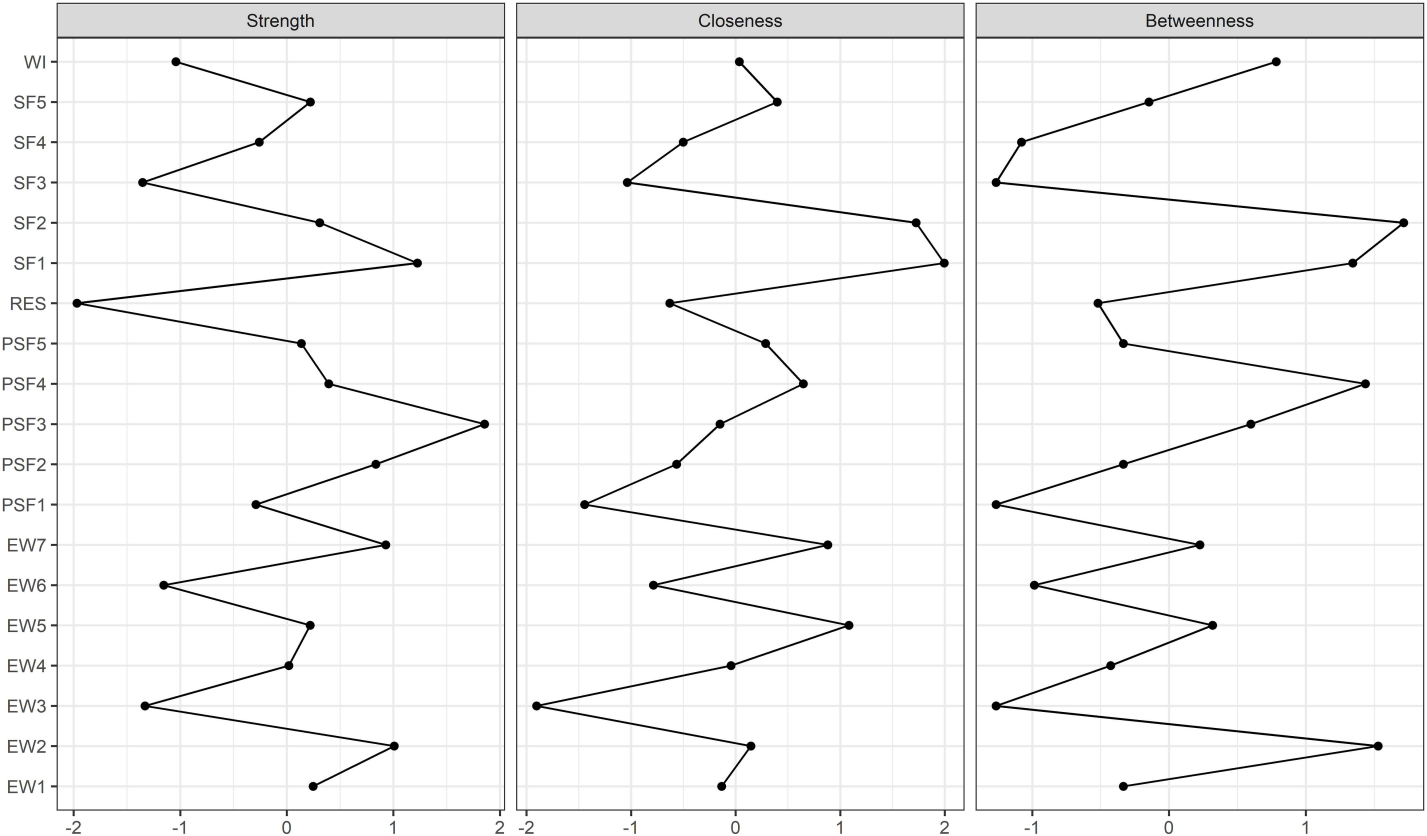
Centrality indices. Centrality indices are presented as standardized *z*-scores. The greater the *z*-score, the more central the factor is.

**Figure 3:**
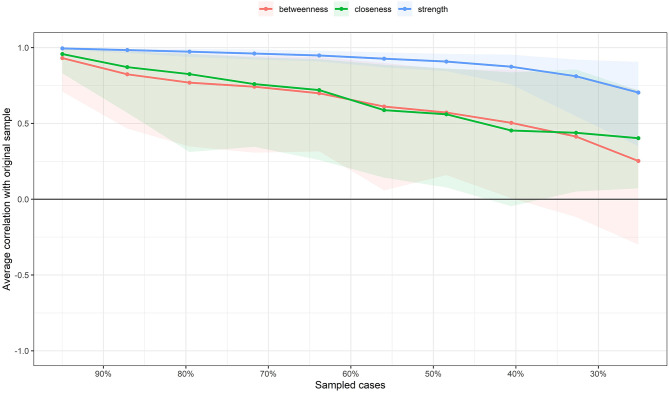
Average correlations between centrality indices of networks sampled with persons dropped and the original sample. Line represents the means and area indicates the range from the 2.5th quantile to the 97.5th quantile.

Centrality indices of node strength, betweenness, and closeness of the estimated network are shown in Figure 2. According to the correlation stability coefficient (Figure 3), the strength centrality index was considered stable, while betweenness and closeness showed poor stability. Therefore, we only interpreted the strength centrality index. The nodes with the highest standardized strength centrality were tremors (PSF3) and anxiety when people coughing (SF1). The bootstrapped difference test for centrality indices indicated that these two nodes had a significantly higher strength (connections) than most other nodes in the network (see Supplementary Figure 3).

## Discussion

In the present study, we investigated the network configuration and centrality indices of Covid-19 fear-related symptoms, work-related stress symptoms, psychological well-being, and resilience in forensic healthcare workers. Overall, the findings suggest that some associations are stronger than others. In general, associations between symptoms within each cluster were higher than associations between clusters. Lastly, we identified the two most central symptoms belonging to the cluster of the Covid-19 fear-related symptoms. This indicates that these symptoms are more influential in this network than the others are.

### Network Configuration

#### Interrelations Between Resilience, Psychological Well-Being, Work-Related Stress and Covid-19 Fear

Consistent with our expectations, psychological well-being was negatively associated with Covid-19 fear-related psychosomatic and work-related stress symptoms, and positively associated with resilience. In particular, we found that workers who have difficulty falling asleep and perceive their work as emotionally demanding reported lower psychological well-being. In contrast, those with higher levels of resilience reported greater psychological well-being. According to the Jobs Demand Resource Model (50), workers who have insufficient personal resources to meet the demands of the job may experience a variety of negative consequences, such as burnout and reduced psychological well-being. As mentioned previously, forensic healthcare workers are at greater risk of being victimized by patients and are therefore in a constant state of alertness at the workplace [e.g., (2)]. However, for some workers, job requirements in this highly stressful and dangerous working environment may exceed their ability to respond appropriately. This, in turn, can lead to exhaustion, which can be manifested in having difficulty falling asleep and perceiving a job as emotionally draining, ultimately leading to diminished psychological well-being. The current study supports previous findings showing that workplace stress and the fear associated with the coronavirus pandemic can negatively affect psychological well-being (26, 28, 51). Conversely, we found that workers who are more resilient, or in other words, more capable of dealing effectively with work pressures and demands, had better psychological well-being. This is consistent with previous research that showed a strong positive link between resilience and well-being (23, 52, 53). Some researchers have argued that greater well-being can serve as an antecedent of resilience, as positive emotions promote flexible thinking, adaptive coping and the maintenance of social relationships (e.g., (54)]. In contrast, others have suggested that the link between well-being and resilience can be also reversed, with resilience being considered a predictor of many well-being outcomes, such as job satisfaction and subjective well-being (55, 56). Although we also found a positive link between resilience and well-being, this cross-sectional and undirected network does not allow us to draw conclusions about the directionality of paths between nodes.

Furthermore, in support of the argument that resilience can serve as a “buffer” against workplace stress (23), we also found that forensic healthcare workers with higher levels of resilience perceive fewer personal attacks or threats at the workplace from patients. This is in line with our hypothesis that resilience would be negatively associated with work-related stress symptoms. Resilient workers may appraise potentially stressful situations as less threatening and reduce distress because of their ability to think flexible and apply adaptive coping strategies (18–22). Last but not least, the current study showed that workers who have contact with difficult clients or patients, are less likely to actively avoid sneezing people. In order to successfully deal with challenging clients or patients, one might need to adopt a more flexible approach when interacting with them. As a result, the worker might become more proficient in social interactions and perhaps less fearful of interacting with others during the Covid-19 pandemic, even with those who sneeze.

Taken together, these findings suggest that the fear due to the Covid-19 pandemic and the symptoms caused by workplace stress can negatively influence workers' well-being. However, resilience might serve as a protective factor for forensic workers against patient-to-worker verbal and physical threats.

#### Connections Within Covid-19 Fear and Work-Related Stress Symptoms

Considering the Covid-19 fear-related psychosomatic symptoms, we found that stomachache, chest pain, and tremors were strongly positively interconnected. This means that forensic healthcare workers who experience stomachaches out of the fear of the coronavirus are also more likely to experience Covid-19 fear-related chest pain and tremors. In addition, we also found a moderately strong and positive association between sleeping problems and tension. The notion that higher levels of tension make it more difficult to fall asleep is empirically supported by many previous studies [e.g., (57, 58)]. However, the opposite holds as well, meaning that a lack of good sleep can contribute to more tension, creating a vicious cycle [e.g., (59)].

Regarding the Covid-19 fear-related social symptoms, the resulting network showed that forensic healthcare workers who feel more anxious when seeing other people coughing are also more likely to actively avoid people who sneeze, and experience greater fear of catching the coronavirus. Likewise, those who become anxious when seeing other people coughing may also be likely, albeit to a lesser extent, to wash their hands very often and have a fear that the coronavirus hinders their social relationship.

Knowledge about the psychological consequences of this pandemic is scarce and there are only a limited number of studies addressing the psychosomatic and social impact of Covid-19 on individual and public health (60). However, the current study largely supports the previous finding showing that fear related to Covid-19 leads to adverse emotional and behavioral outcomes, such as loneliness, anxiety and sleep problems (61). However, this cross-sectional network does not allow us to draw conclusions about the (causal) direction of pathways between Covid-19 fear-related symptoms. Nonetheless, it offers insight into which of these symptoms tend to co-occur and which connections are important for forensic healthcare workers. These findings may be highly relevant in clinical practice for developing strategies to combat the fear posed by the Covid-19 pandemic.

In addition, the current study also contributes to understanding the patterns of work-related stress symptoms. In particular, we found that forensic healthcare workers who experience their work as more emotionally demanding are also more likely to be confronted with matters that affect them personally. Likewise, workers who have more contact with difficult patients are more likely to end up in harrowing situations at the workplace. The results further showed that workers who are confronted in their work with matters that affect them personally are also more often approached by others for personal matters. Finally, those who have contact with difficult patients may also experience that their work requires more persuasion. In short, this study reveals which work-related stress symptoms tend to occur together. This knowledge could be useful for developing stress management interventions at the workplace.

### Network Centrality

In accordance with our hypothesis, Covid-19 fear-related psychosomatic and social symptoms were centrally embedded in the network. Specifically, we found that tremors due to the fear of the coronavirus and anxiety when other people coughing were the most central symptoms in the network. It is well-known that cough represents one of the most common clinical features of Covid-19. In fact, large droplets generated during coughing can transmit the infection very easily (62). Hence, it is not surprising that seeing and hearing people coughing during this global pandemic can cause considerable stress and potentially evolve other fear and stress related symptoms. Likewise, it is widely recognized that tremor or shaking is a common symptom in people with anxiety, which can be very disturbing and cause significant amounts of stress (63). In line with this, the current study showed that the tremor can indeed activate other symptoms, as it was strongly connected to many different symptoms in the network (64). Furthermore, because the fear caused by the Covid-19 outbreak can have drastic negative effects on peoples' mental health, it has recently been proposed to classify this persistent and excessive fear of the coronavirus as a certain type of *DSM-5* specific phobia (26). In addition, our research also revealed which coronavirus fear-related symptoms tend to occur together, which can also be of great value when determining a diagnosis.

### Limitations

Despite its strengths, some limitations of this study must be acknowledged. First, the study sample consists only of forensic healthcare workers who are in direct contact with patients, and therefore the results cannot be generalized to those in forensic psychiatry who do work directly with patients, such as secretarial staff and supporting managers. Another limitation is that we relied on self-report data that may confound the results through reporting bias. In addition, the response rate was lower than expected, potentially leading to sampling bias. Further studies with larger sample sizes are needed to confirm our findings. The study was also limited by the fact that we have no information if and how many workers and patients were infected with coronavirus in the period leading up to the survey. Infection of workers and/or patients can influence the study results because when more people are infected with Covid-19, the fear is likely to be greater. Finally, this cross-sectional network model does not allow us to draw any conclusions about the potential causal nature of connections between nodes. Therefore, longitudinal network designs are needed to further investigate which symptoms can cause or trigger each other causally.

In conclusion, this was the first study investigating a network configuration of Covid-19 fear-related symptoms, work-related stress symptoms, resilience and psychological well-being in forensic healthcare workers. The results of this study are consistent with existing research demonstrating that workplace stress can be detrimental to workers' psychological well-being and that resilience can serve as a protective factor against being personally attacked at the workplace. In addition, we found that the fear associated with the ongoing Covid-19 pandemic negatively affects the psychological well-being of forensic healthcare workers. This knowledge is highly relevant in clinical practice for designing timely interventions for reducing workplace stress and fear due to the pandemic. In addition, we identified highly central symptoms that would be the best candidates for future treatment targets. Although this work prevents us from making causal claims, it certainly offers a valuable first step in shaping future longitudinal research.

## Data Availability Statement

The raw data supporting the conclusions of this article will be made available by the authors, without undue reservation.

## Ethics Statement

The studies involving human participants were reviewed and approved by the Scientific Research Committee of Fivoor, the Netherlands. The patients/participants provided their written informed consent to participate in this study.

## Author Contributions

SB and MJ analyzed the data. SB wrote the first draft of the manuscript. MJ, MW, YE, ED, CG, IF, IJ, and EM critically revised the manuscript for important intellectual content. All authors contributed to the article and approved the submitted version.

## Conflict of Interest

The authors declare that the research was conducted in the absence of any commercial or financial relationships that could be construed as a potential conflict of interest.
