# Supplementary material for: Associations Between Resilience, Psychological Well-Being, Work-Related Stress and Covid-19 Fear in Forensic Healthcare Workers Using a Network Analysis
Source: Front Psychiatry. 2021 Jun 11;12:678895. doi: 10.3389/fpsyt.2021.678895 (PMC8226029; doi:10.3389/fpsyt.2021.678895)
Supplement: Supplementary file 1 [file Data_Sheet_1.docx]

Supplementary Material

# Supplementary Tables

**Table S1**

Variables Used in Network Analysis

| WI “Well-being index” |
| --- |
| RES “Resilience” |
| PSF1 “I experience severe stomachaches because of coronavirus.” |
| PSF2 “I experience serious chest pain because of coronavirus.” |
| PSF3 “I experience tremors due to coronavirus.” |
| PSF4 “I experience sleep problems because of coronavirus.” |
| PSF5 “Coronavirus makes me so tense that I cannot even do the things I regularly can.” |
| SF1 “After coronavirus pandemic, I feel extremely anxious when I see people coughing.” |
| SF2 “After coronavirus pandemic, I run away from people sneezing.” |
| SF3 “After coronavirus pandemic, I notice that I spend too much time for cleaning my hands.” |
| SF4 “Fear of coming down with coronavirus seriously impedes my social relationships.” |
| SF5 “I cannot refrain my anxiety of catching coronavirus from others.” |
| EW1 “Is your job emotionally demanding?” |
| EW2 “Are you confronted in your work with things that affect you personally?” |
| EW3 “Are you personally called on by others in your work?” |
| EW4 “Do you feel personally attacked or threatened in your work?” |
| EW5 “Do you have contact with difficult clients or patients in your work?” |
| EW6 “Does your job require persuasion?” |
| EW7 “Does your work put you in harrowing situations?” |

*Note.* WI=Well-being index; RES=Resilience; PSF=Psychosomatic Covid-19 fear related symptoms; SF=Social Covid-19 fear related symptoms; EW=Work-related stress symptoms.

Table S2

Correlations Among Study Variables

|  | 1 | 2 | 3 | 4 | 5 | 6 | 7 | 8 | 9 | 10 | 11 | 12 | 13 | 14 | 15 | 16 | 17 | 18 | 19 |
| --- | --- | --- | --- | --- | --- | --- | --- | --- | --- | --- | --- | --- | --- | --- | --- | --- | --- | --- | --- |
| 1. WI | - |  |  |  |  |  |  |  |  |  |  |  |  |  |  |  |  |  |  |
| 2. RES | .289^**^ | - |  |  |  |  |  |  |  |  |  |  |  |  |  |  |  |  |  |
| 3. SF1 | -.166^**^ | -.045 | - |  |  |  |  |  |  |  |  |  |  |  |  |  |  |  |  |
| 4. SF2 | -.051 | -.003 | .624^**^ | - |  |  |  |  |  |  |  |  |  |  |  |  |  |  |  |
| 5. SF3 | .005 | .083 | .423^**^ | .359^**^ | - |  |  |  |  |  |  |  |  |  |  |  |  |  |  |
| 6. SF4 | -.143^*^ | -.072 | .540^**^ | .442^**^ | .386^**^ | - |  |  |  |  |  |  |  |  |  |  |  |  |  |
| 7. SF5 | -.178^**^ | -.094 | .605^**^ | .476^**^ | .314^**^ | .536^**^ | - |  |  |  |  |  |  |  |  |  |  |  |  |
| 8. PSF1 | -.205^**^ | -.008 | .396^**^ | .235^**^ | .208^**^ | .323^**^ | .387^**^ | - |  |  |  |  |  |  |  |  |  |  |  |
| 9. PSF2 | -.190^**^ | -.038 | .373^**^ | .216^**^ | .169^**^ | .251^**^ | .427^**^ | .718^**^ | - |  |  |  |  |  |  |  |  |  |  |
| 10. PSF3 | -.179^**^ | -.032 | .434^**^ | .252^**^ | .212^**^ | .258^**^ | .437^**^ | .738^**^ | .841^**^ | - |  |  |  |  |  |  |  |  |  |
| 11. PSF4 | -.406^**^ | -.121^*^ | .423^**^ | .243^**^ | .228^**^ | .361^**^ | .400^**^ | .478^**^ | .546^**^ | .510^**^ | - |  |  |  |  |  |  |  |  |
| 12. PSF5 | -.251^**^ | -.093 | .394^**^ | .204^**^ | .260^**^ | .376^**^ | .372^**^ | .523^**^ | .544^**^ | .561^**^ | .551^**^ | - |  |  |  |  |  |  |  |
| 13. EW1 | -.223^**^ | -.152^**^ | .141^*^ | .044 | .062 | .090 | .143^*^ | .125^*^ | .094 | .101 | .173^**^ | .186^**^ | - |  |  |  |  |  |  |
| 14. EW2 | -.117^*^ | -.118^*^ | .213^**^ | .229^**^ | 0.080 | .119^*^ | .136^*^ | .191^**^ | .173^**^ | .147^**^ | .164^**^ | .176^**^ | .528^**^ | - |  |  |  |  |  |
| 15. EW3 | -.163^**^ | -.056 | .098 | .030 | .052 | .077 | .112^*^ | .096 | .060 | .031 | .133^*^ | .103 | .325^**^ | .390^**^ | - |  |  |  |  |
| 16. EW4 | -.213^**^ | -.234^**^ | .061 | .035 | .077 | .191^**^ | .087 | .171^**^ | .156^**^ | .151^**^ | .181^**^ | .195^**^ | .395^**^ | .430^**^ | .317^**^ | - |  |  |  |
| 17. EW5 | -.123^*^ | -.087 | -.011 | -.128^*^ | .015 | .025 | .016 | .042 | .093 | .074 | .106 | .157^**^ | .383^**^ | .193^**^ | .234^**^ | .278^**^ | - |  |  |
| 18. EW6 | -.089 | .003 | .111 | .085 | .061 | .145^*^ | .124^*^ | .116^*^ | .163^**^ | .177^**^ | .145^*^ | .202^**^ | .253^**^ | .250^**^ | .228^**^ | .253^**^ | .373^**^ | - |  |
| 19. EW7 | -.070 | -.016 | .091 | .031 | .082 | .122^*^ | .086 | .040 | .074 | .019 | .083 | .148^**^ | .434^**^ | .414^**^ | .240^**^ | .372^**^ | .506^**^ | .412^**^ | - |

*Note*. ** Correlation is significant at the .01 level (2-tailed); * Correlation is significant at the .05 level (2-tailed). WI=Well-being index; RES=Resilience; SF=Social Covid-19 fear related symptoms; PSF=Psychosomatic Covid-19 fear related symptoms; EW=Work-related stress symptoms. For full variable names, see Table S1 in the supplementary material.

# Supplementary Figures


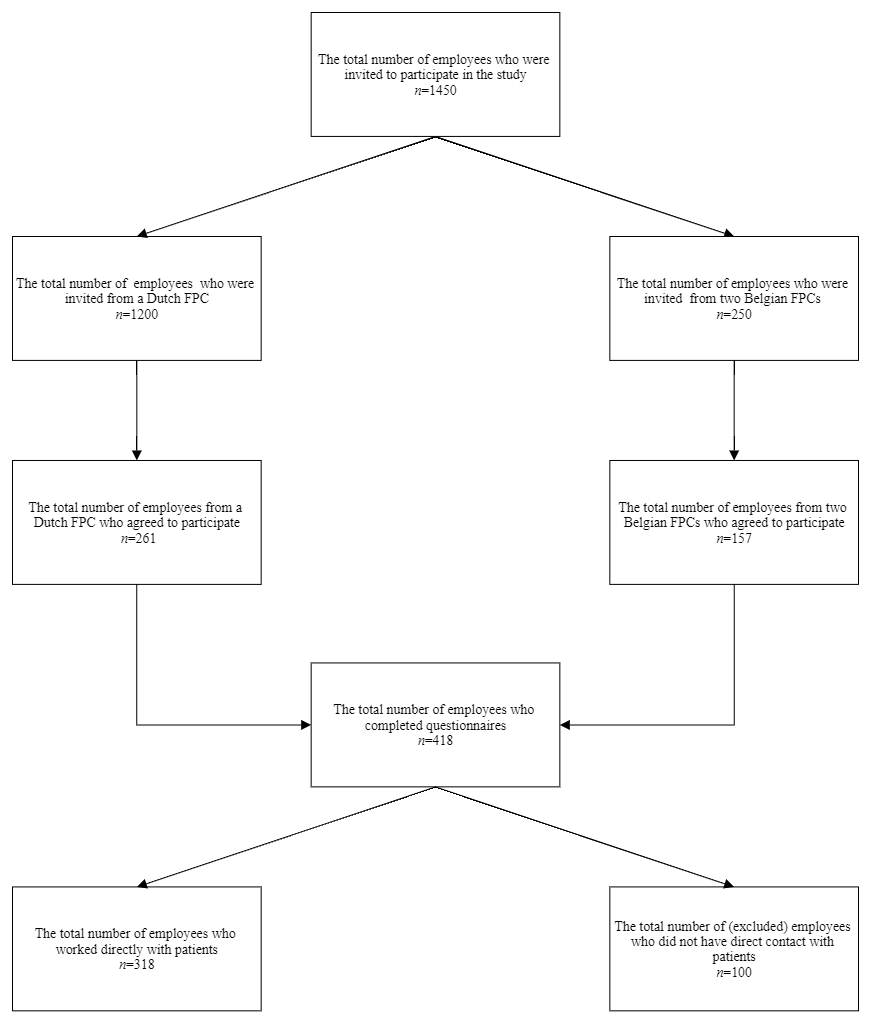


Figure S1. An overview of the recruitment process. FPC=Forensic psychiatric center.


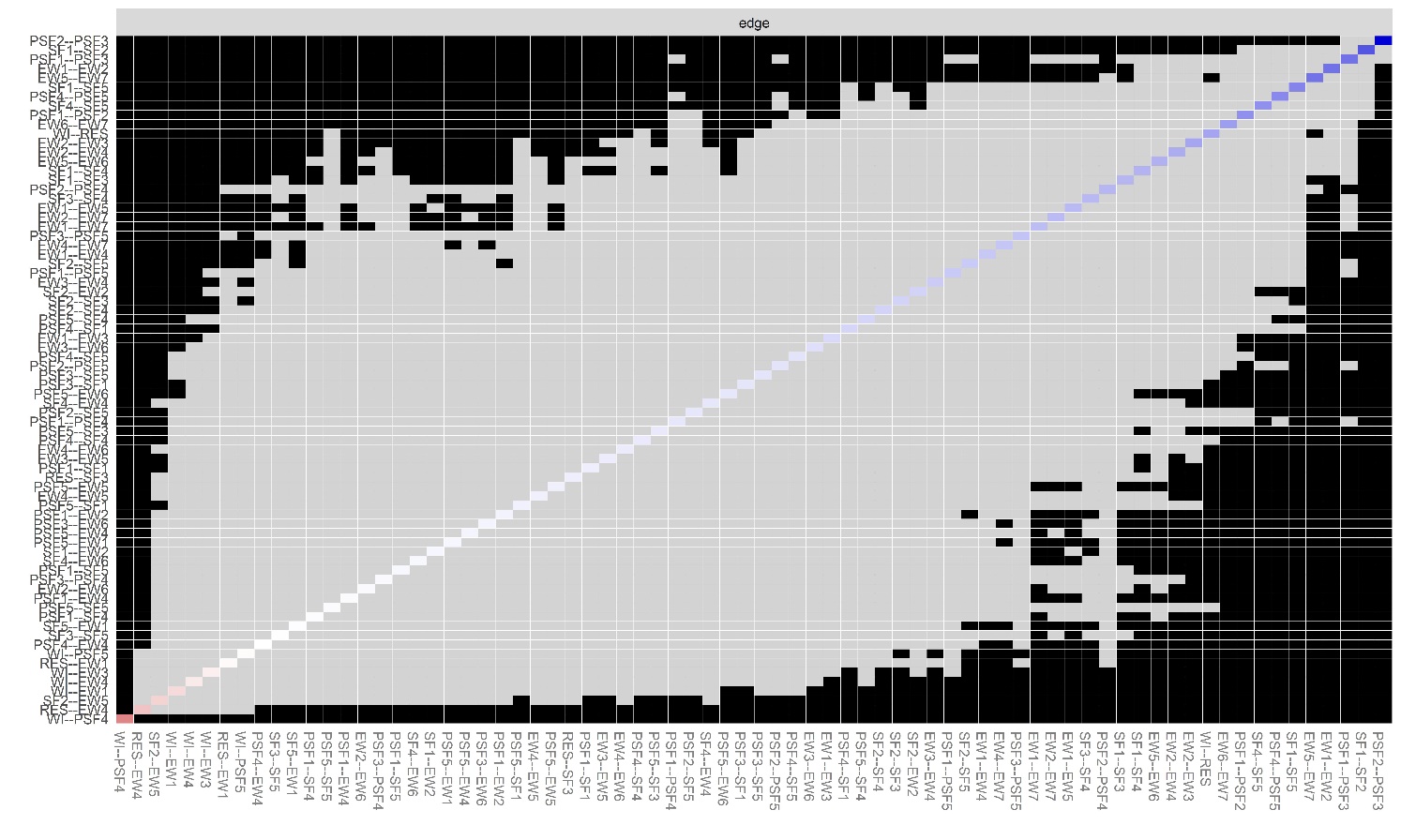


Figure S2. Bootstrapped Edge Weights Difference Test. The gray boxes represent edges that do not differ significantly from one-another and black boxes represent edges that do differ significantly from one-another.


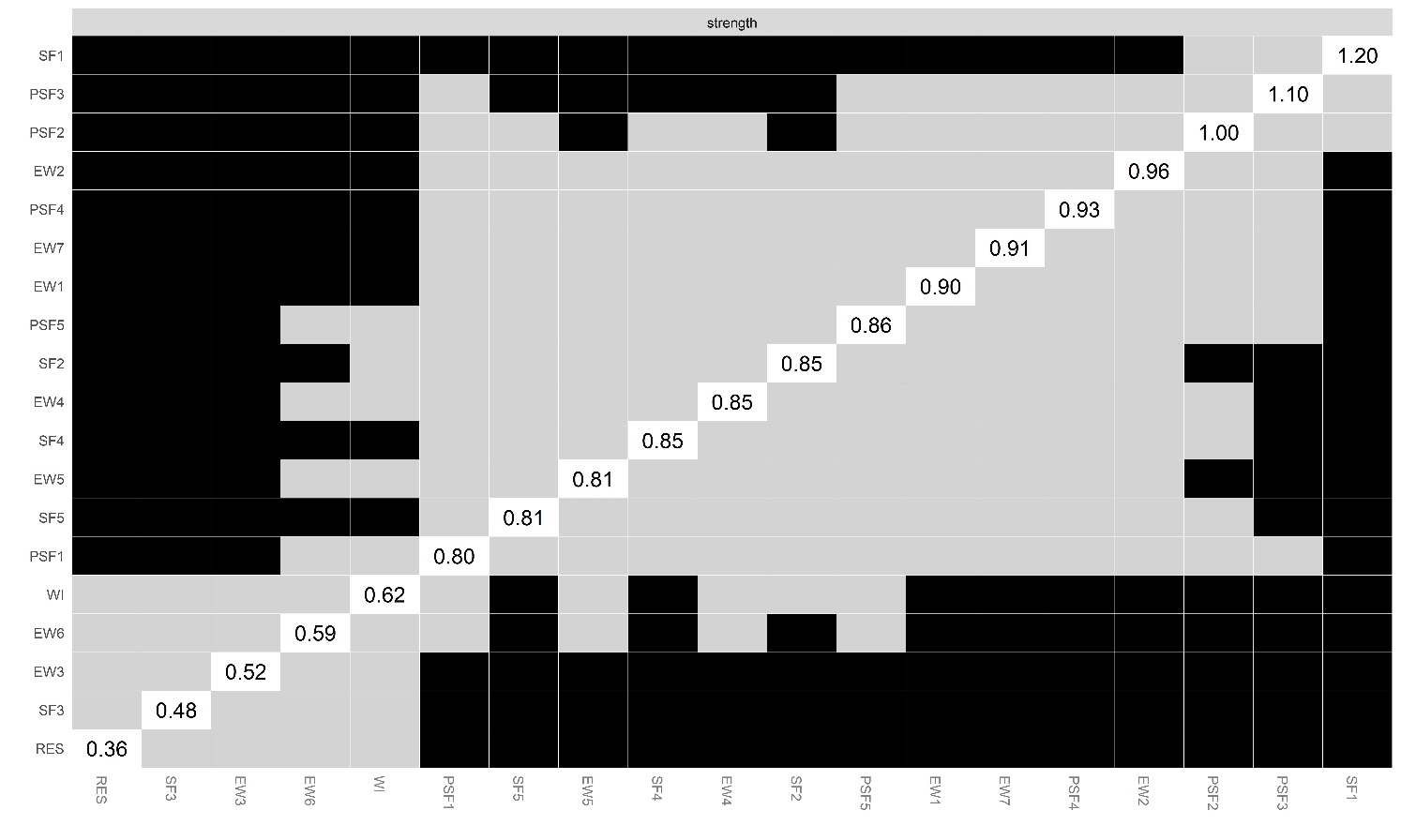


**Figure S3. Bootstrapped Strength Centrality Difference Test.** The gray boxes represent nodes that do not differ significantly from one-another and black boxes represent nodes that do differ significantly from one-another. The white boxes show the value of node strength.
